# An Uncommon Case of Plasma Cell Mucositis of the Tongue in a Young Man

**DOI:** 10.1155/2020/3429632

**Published:** 2020-02-18

**Authors:** Alessandro Antonelli, Fiorella Averta, Federica Diodati, Danila Muraca, Ylenia Brancaccio, Chiara Mignogna, Amerigo Giudice

**Affiliations:** ^1^School of Dentistry, Department of Health Sciences, “Magna Graecia” University of Catanzaro, Italy; ^2^Interdepartmental Service Center, “Magna Graecia” University of Catanzaro, Italy

## Abstract

Plasma cell mucositis (PCM) is an unusual plasma cell proliferative disorder of the upper aerodigestive tract. It is a rare disease, and its etiology is not yet known with variable clinical features. Symptoms include dysphagia, oral pain, and swelling. We described a case of PCM involving the tongue of a 14-year-old man. In the first place, several diagnostic hypotheses were proposed, most of them discarded for incompatibility with blood and laboratory tests. This disease rarely manifests itself on the tongue, especially in young patients with no comorbidities. The management of PCM is mainly aimed at reducing the symptoms, and in our report, the treatment involved the use of systemic prednisone with an improvement of the quality of life. At 1-year follow-up, there was no recurrence of the disease. Many therapeutic treatments are able to stabilize but not able to induce a complete remission. PCM is considered an uncommon benign disorder with a favorable prognosis and should be considered in the differential diagnosis with other inflammatory or neoplastic conditions.

## 1. Introduction

Plasma cell mucositis (PCM) is an unusual plasma cell proliferative disorder of the upper aerodigestive tract [1]. In the past, this pathology has been reported under various names. The name indicated the anatomical structure involved together with the component of plasma cells, such as idiopathic plasmacytosis of the gingiva, plasma cell vulvitis, oral papillary plasmacytosis, or mucous membrane plasmacytosis of the upper aerodigestive tract [2]. It is a rare disease, and its etiology is not yet known; it is considered a benign condition of adults, and there are no correlations in literature with the development of plasma cell neoplasm. Clinical features are an intensely erythematous mucosa with papillomatous, cobblestone, nodular, or velvety surface changes. Symptoms include dysphagia, oral pain, sore throat, and pharyngitis [2]. PCM can be treated with corticosteroids, administrated topically, systemically, or intralesionally. Usually, the treatment is not resolutive; however, the main outcome is the improvement of the symptoms. Generally, PCM patients have a previous history of an autoimmune or immunologically mediated disease like Sjögren syndrome or possible autoimmune hepatitis; however, these are not present in all cases and no single disease is consistently associated.

## 2. Case Report

We described a case of PCM involving the tongue of a 14-year-old man. The patient was referred to the Oral Pathology Unit at the Faculty of Dentistry, Magna Graecia University of Catanzaro, in August 2019. The referring practitioner suspected squamous cell carcinoma. His past medical history was not relevant, and the patient was a nonsmoker and nonalcohol user. The patient had previously used a nocturnal bite to control bruxism. He reported a burning sensation in his mouth, local dysgeusia, and pain on the tip and on the right lingual border for 1 year. The patient also referred that he was unable to eat or drink any hot or spicy foods due to burning and pain on his tongue. He stated persistent hoarseness, sore throat, and difficulty sleeping due to continuous oral pain.

**Figure 1:**
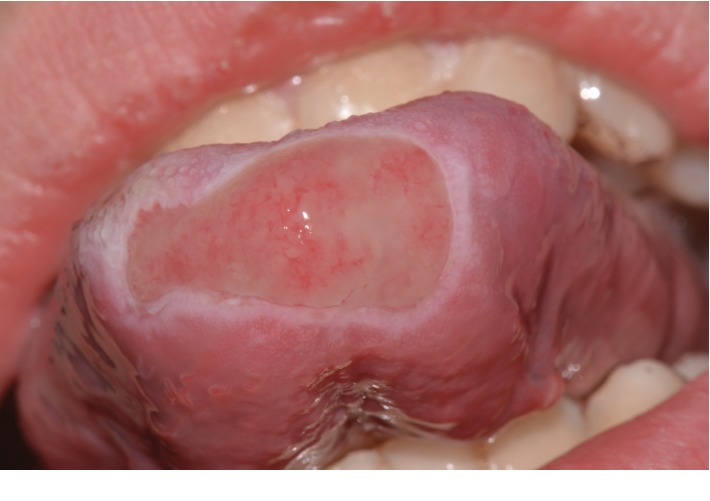
Ulceration on the tip of the tongue.

**Figure 2:**
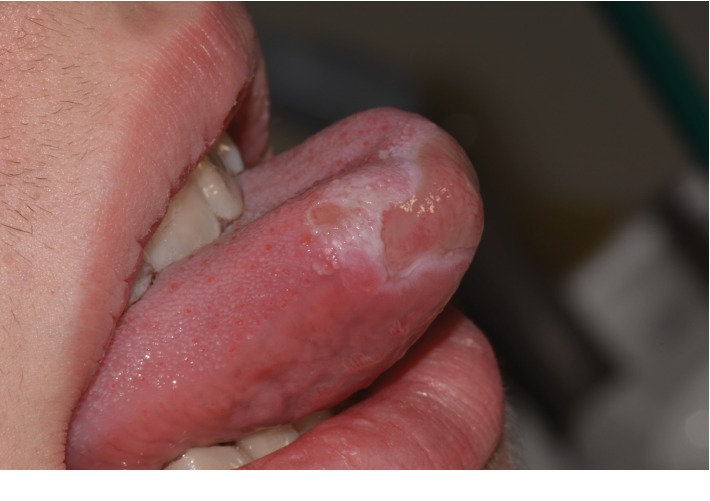
Lateral view of the tongue.

**Figure 3:**
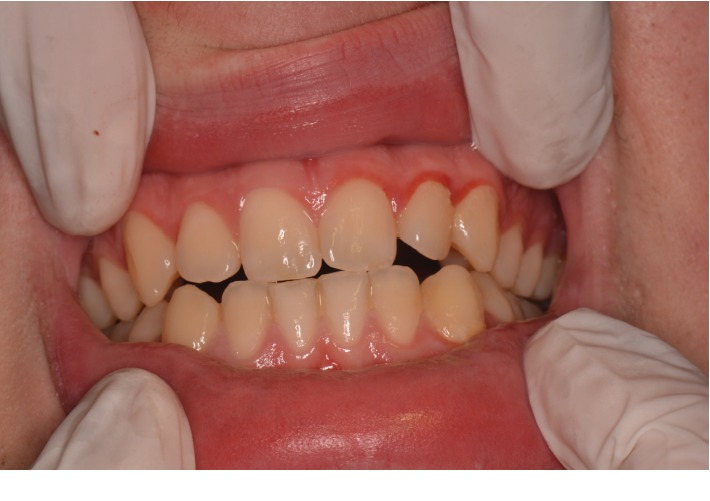
Gingival edema and erythema.

**Figure 4:**
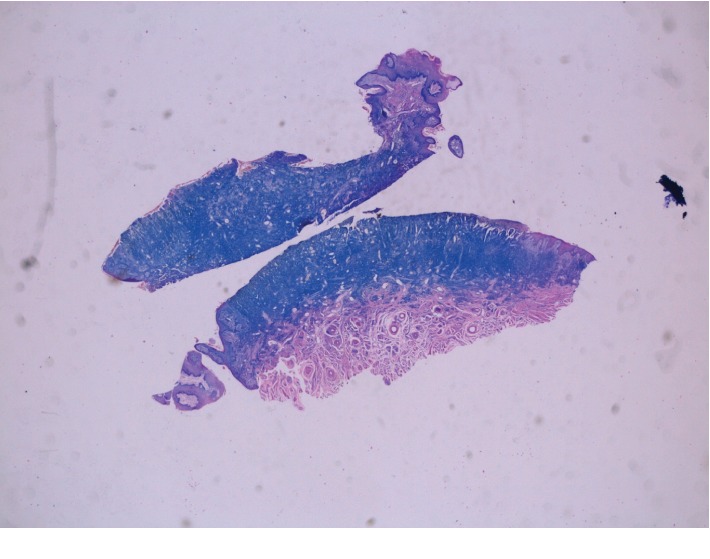
Medium-power hematoxylin and eosine-stained biopsy sample from the tongue mucosa showing a dense polyclonal plasmacytic inflammatory infiltrate throughout the connective tissue.

**Figure 5:**
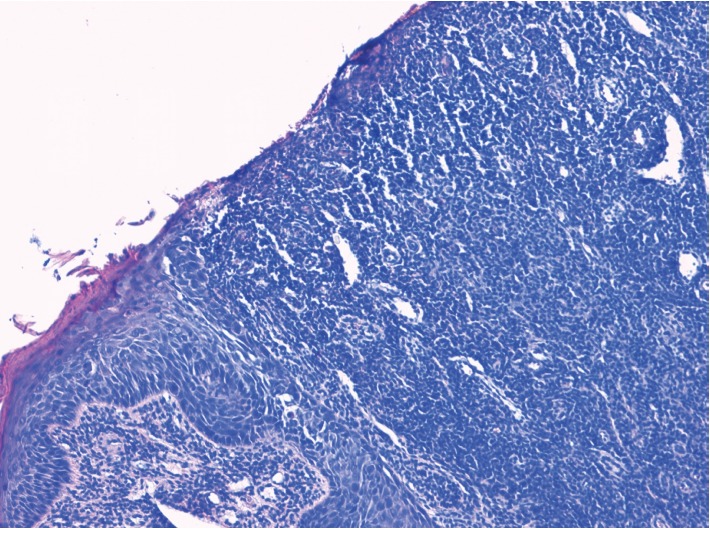
Histological examination of a biopsy taken from the tongue showed a denuded surface epithelium with the superficial and mid dermis demonstrating a dense infiltration of plasma cells.

**Figure 6:**
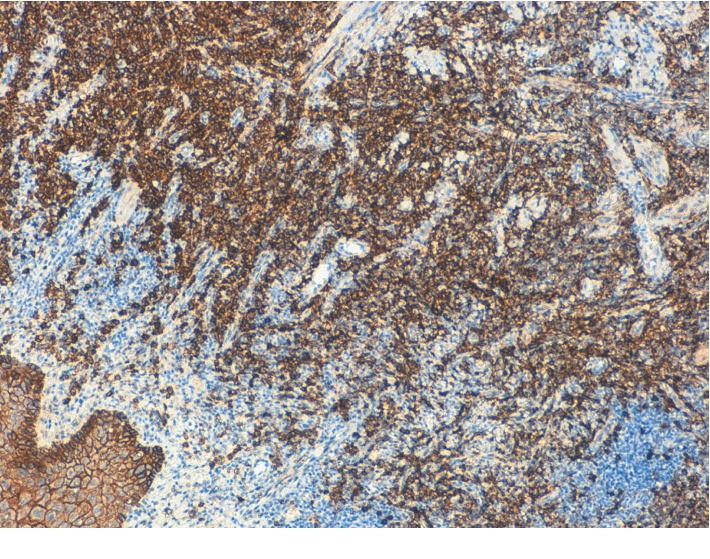
CD138. A high-power view shows a monomorphic population of mature plasma cells (×10).

**Figure 7:**
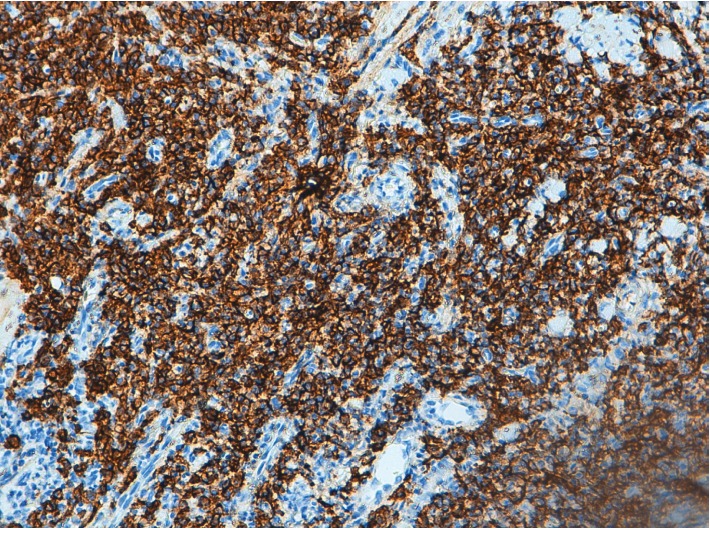
CD138. A high-power view shows a monomorphic population of mature plasma cells (×20).

**Figure 8:**
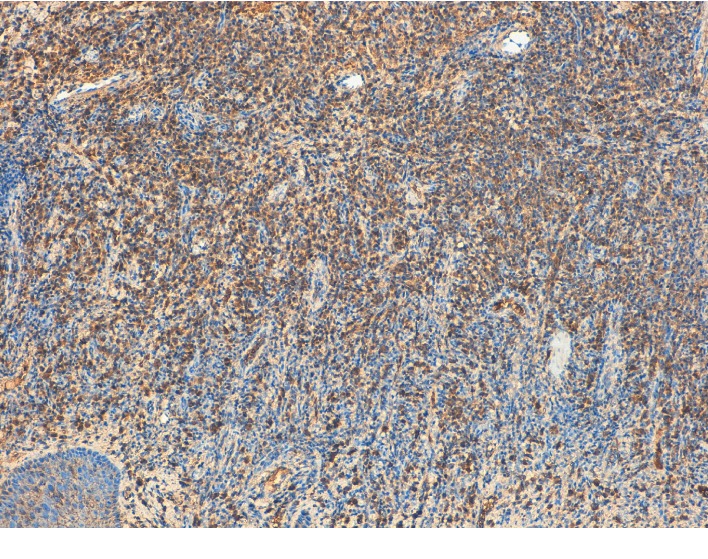
Plasma cells showing kappa light chain restriction (×10).

**Figure 9:**
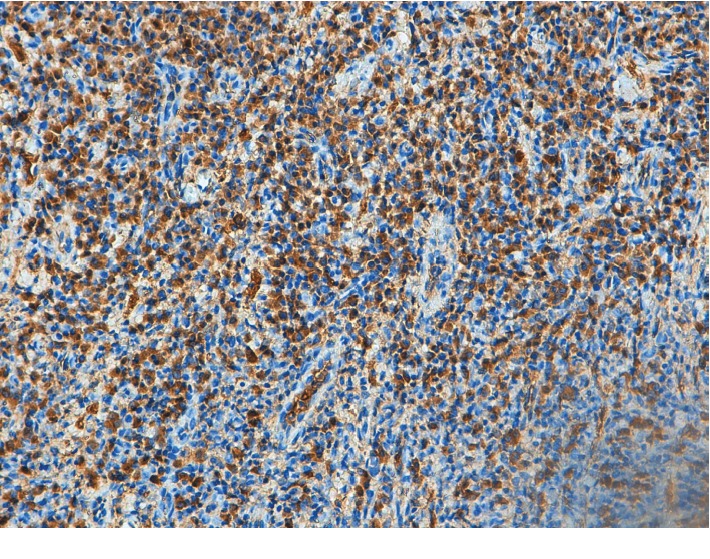
Plasma cells showing kappa light chain restriction (×20).

**Figure 10:**
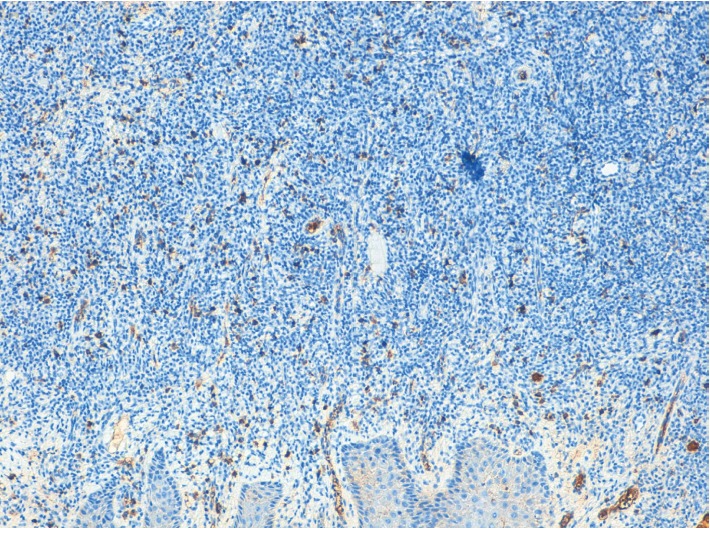
Plasma cells showing lambda light chain restriction (×10).

**Figure 11:**
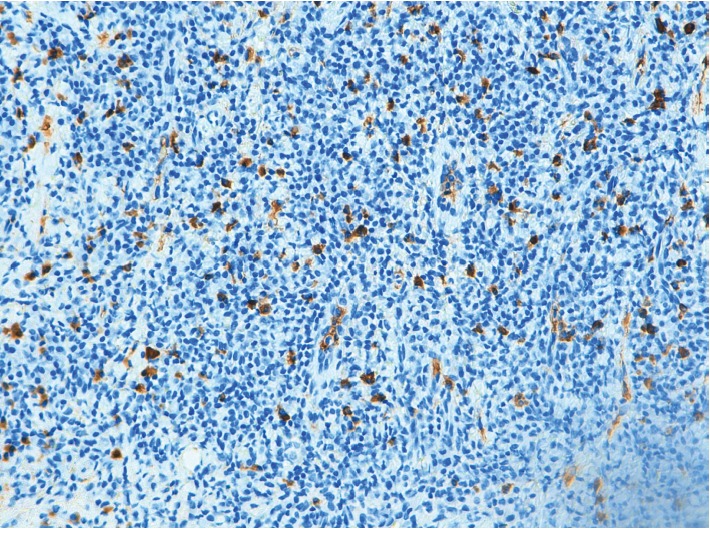
Plasma cells showing lambda light chain restriction (×20).

**Figure 12:**
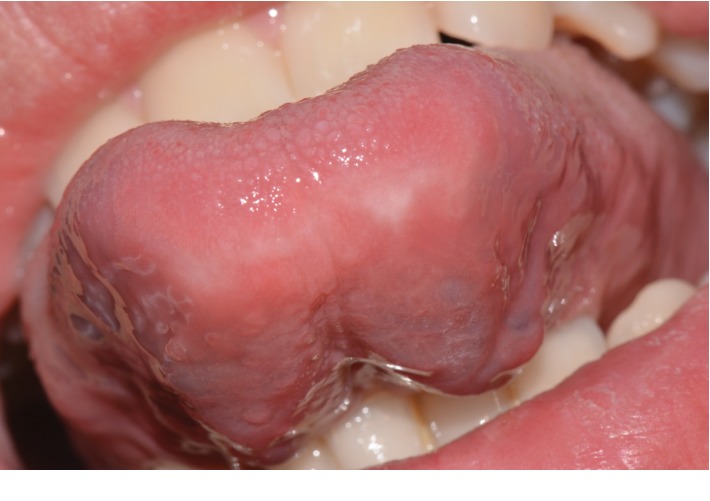
Follow-up at 1 year.

Extraoral examination was unremarkable, while intraoral examination revealed the presence of an ulcer and intense erythema of the tongue (Figures 1 and 2) and gingival edema and erythema (Figure 3); however, the other parts of the oral mucosa was clinically normal. In the first place, several diagnostic hypotheses were proposed, most of them discarded for incompatibility with full blood count, serum B12 and folate, urea and electrolytes, liver function tests, glucose, anti-nuclear antibody, and syphilis IgG that were normal or negative. The patient underwent an incisional biopsy under local anesthesia. The specimen was stored in a tube containing formalin 10% and sent to a laboratory for histopathological analysis. Microscopically, a large area of ulceration of the coating epithelium subtended by dense plasma cell infiltrate was observed (Figures 4 and 5). CD20 and CD3 showed focal positivity for B and T cells, respectively. The plasma cell infiltrate was positive for CD138 (Figures 6 and 7) and showed kappa light chain restriction (Figures 8 and 9) with a kappa/lambda ratio of 20 : 1 (Figures 10 and 11). The final histopathological diagnosis was “plasmacytosis of the mucous membranes with restriction for the kappa chains.” Initial pharmacotherapy with prednisone began with 50 mg/day for two weeks, and the patient referred an improvement of his conditions (reduction of pain and swelling). He repeated this treatment one month later, and he experienced a partial regression of the lesions. At 1-year follow-up, there was no regression of the disease and no recurrence of the lesions (Figure 12).

## 3. Discussion

Plasma cell mucositis (PCM) is an idiopathic disorder histologically characterized by dense infiltrates of lymphocytes and plasma cells in submucosa. Usually, the lesions are mainly observed on the mucosal surfaces, vulva and penis, gingiva, lips, tongue, buccal mucosa, epiglottis, and larynx.

**Figure 13:**
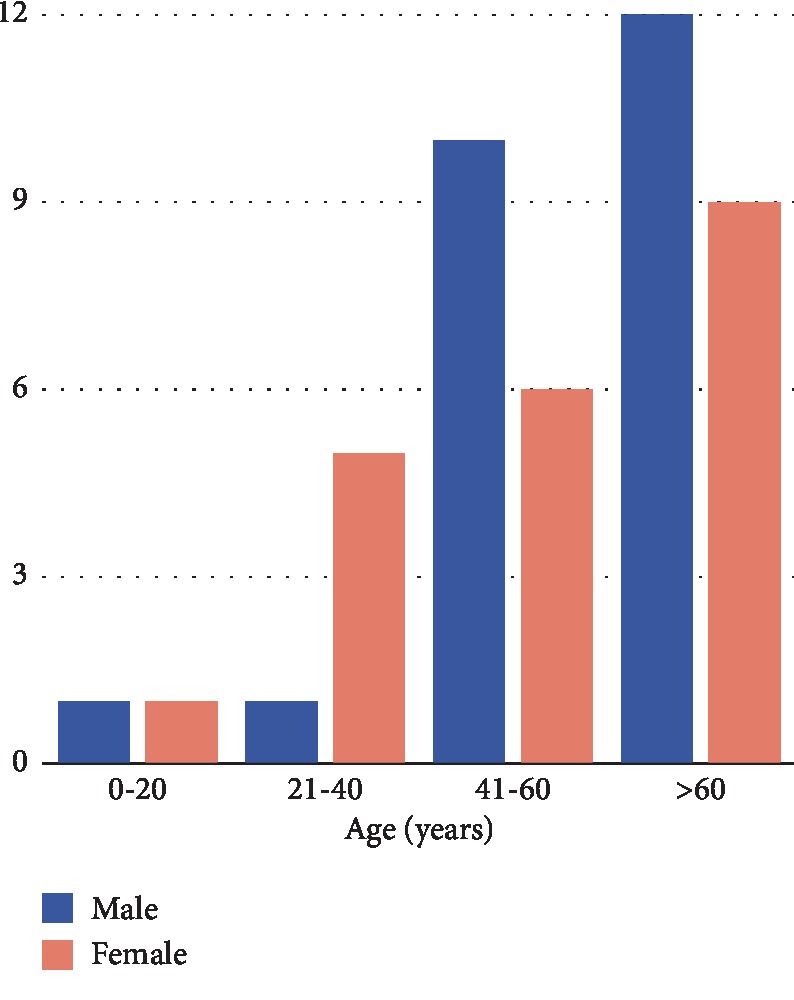
Distribution of age and sex in the 45 cases of PCM of the oral cavity reviewed.

A review of the English-language literature was performed. The keywords “plasma cell mucositis” and “oral” were entered in the search fields of PubMed. 103 results were identified. Another research was performed including the keywords “plasmacytosis” and “oral.” 40 results were identified. The research was conducted by considering the articles published until November 2019. Excluded were cases that were not about the oral cavity, not in English, not human study, and not compatible with the PCM diagnosis or report lacking immunohistochemical analysis. According to the exclusion criteria, we selected 26 studies [1–26] that included 45 cases of PCM. We analysed patients' age, gender, symptoms, lesion location, treatment, and follow-up. The principal features and data pertaining the selected cases are compiled in Table 1. Age data were available in all selected studies, and the average age was 56.06 ± 16.33 years. The youngest patient was 13 years old [17] and the oldest 83 years old [20]. All studies included in our review reported the gender of the subjects. The analysis of the data collected suggested a male predilection with a male-to-female ratio of 1.25 : 1 (45 cases: 25 M, 20 F). A graph of the age and gender distribution is reported in Figure 13. Rarely the lesions are isolated, and in 45 cases reviewed, the oral cavity zones most affected by the lesions were the gingiva (20 cases), the lips (14 cases), the upper aerodigestive tract (13 cases), the palate (12 cases), the buccal mucosa (11 cases), and the tongue (8 cases). In our review, the symptoms most present are typical of inflammation with a particular interest of the oropharynx; the main symptoms are erythema (13 cases), pain (10 cases), swelling (9 cases), sore throat (7 cases), dysphonia, and dysphagia (6 cases). The analysis of the treatment evidenced 24 cases (53.3%) treated with corticosteroids in different forms (systemic, topical, and injections), and in 10 cases of these, there was no recurrence of the disease.

Plasma cell mucositis (PCM) is classified as idiopathic and rarely manifests itself on the tongue, especially in young patients with no comorbidities. Despite the fact that plasma cell mucositis often involves the oral and genital mucosa [3], there was no genital involvement in this circumstance. Since it is a very rare pathology, in the diagnosis of PCM, it is important to exclude other pathologies with similar pathological features, such as erosive lichen planus, mucous membrane pemphigoid, sarcoidosis, allergic gingivostomatitis, extramedullary plasmacytoma, rhinoscleroma, pemphigus, erythroplasia, squamous cell carcinoma, and fungal infections [4, 5]. Lichen planus is a chronic-relapsing inflammatory disease, with cutaneous and mucous involvement. It can be distinguished in oral lichen planus (OLP) and oral lichenoid drug reactions (OLDRs). The lesions may cause pain and, generally, appear as white patches with erosive aspects. A typical OLP model is identified with bilateral and symmetrical lesions; moreover, a hyperkeratosis is observed in the lower epidermis and in the upper dermis [27]. Pemphigoid of the mucous membranes affects mainly older individuals, as well as the plasma cell mucositis. Unlike the PCM, it presents itself with erosions to the mucous membranes of variable gravity that are mainly located in the oral cavity [5]. Sarcoidosis is a rare acquired systemic granulomatous disease. The respiratory system is more interested, but in a smaller number of cases, there is the involvement of the oral and perioral mucosa. Oral lesions can be solitary or multiple; they appear as a well-delimited and occasionally ulcerated red swelling [28]. Allergic gingivostomatitis is the result of an immunologic injury reaction; it is a complex disease which can have more than one etiologic factor [29]. Extramedullary plasmacytoma is a tumor that rarely affects the head and neck, especially the upper airways, and the lesions are generally polypoidal in appearance [5]. Primary Non-Hodgkin's Lymphoma (NHL) can rarely arise from lymphoid tissue of the tongue and give manifestations that mimic PCM [30].

Several times, PCM lesions can enter into differential diagnosis with squamous cell carcinoma (SCC) [4–22]; in literature, there are cases in which a differential diagnosis was made with oral carcinoma and there is a case report where a SCC arose from a mucosal plasmacytosis [13]. Therefore, a correct diagnosis and adequate management of the disease are essential.

The management of PCM is mainly aimed at reducing the symptoms; in fact, the patient treatment involved the use of systemic prednisone 50 mg/day to improve his quality of life.

However, in literature, there are some reported cases where the use of PRF injections is associated with cortisone therapy [6]. The benefit of PRF is to allow a continuous release of growth factors, inducing neoangiogenesis and fibroblast activations. Therefore, PRF creates an optimal scaffold for the tissue healing process, with the advantage of low cost and easy to prepare [31].

Although many therapeutic treatments are able to stabilize the disease, they are not able to induce a complete remission. The condition is generally of long-standing duration and the disease has a significant impact on the patient's quality of life. The disease treatment is mainly targeted to the management of symptoms. PCM is considered an uncommon benign disorder with a favorable prognosis. The distinctions between the conditions that are present in the oral cavity with the histologic finding of a dense submucosal plasma cell infiltrate are not well documented in oral pathology literature.

## 4. Conclusion

It is important that PCM is recognized in the dental community, because diagnosis is dependent on clinical pathologic correlation. Only close communication between specialists in several disciplines prevented inappropriate treatment of a patient with an extremely rare condition. Nevertheless, it is very important to differentiate the PCM disease from other neoplastic conditions in order to achieve a better clinical management of the patients, so it is necessary to investigate this disease in depth.

## Figures and Tables

**Table 1:** Review of PCM.

Study/year of publication	Age^∗^	Gender	Symptoms	Location of the lesions	Treatment	Follow-up
Poswillo et al. [7]/1967	37	F	Redness, swelling	Gingiva	Resection, oral hygiene programme	AWD, 2 years
	39	F	Redness, hypertrophy	Gingiva	Resection	Not available
	21	F	Hyperplastic red lesion, hypertrophy, erythema	Maxillary labial vestibule, gingiva	Resection, oral hygiene programme	Not available

White et al. [3]/1986	47	F	Dysphonia, sore throat	Lips, mouth, tongue, supraglottic larynx	Resection, prednisone	AWD, 9 years (f/u reported by Ferreiro et al. [1])

Timms et al. [8]/1988	70	F	Hoarseness, cough, stridor	Gingiva, supraglottic, hard palate	CO_2_ laser, prednisolone, topical steroid spray	AWD, 1 year

Timms and Sloan [9]/1991	32	F	Hoarseness, sore throat	Gingiva, false cord, mucobuccal	Systemic and topical steroids	Marginal improvement, no f/u epiglottis

Ferreiro et al. [1]/1994	60	F	Dysphonia, dysphagia	Supraglottic larynx	CO_2_ laser, prednisone, antibiotics, beclomethasone (Vanceril) spray	AWD, 1 year
	41	M	Dysphonia, stridor	Supraglottic, glottic larynx, nose, pharynx	Antibiotics	AWD, 7 years; tracheostomy
	62	F	Dysphonia, stridor, dry eyes, dysphagia	Supraglottic, glottic larynx, trachea	Not available	AWD, 16 years; tracheostomy
	54	M	Dysphonia	Supraglottic, glottic larynx	Resection	AWD, 1 year
	40	M	Dysphonia, sore mouth	Lips, tongue, palate, pharynx, supraglottic & glottic larynx	Prednisone, isotretinoin (Accutane), CO_2_ laser	AWD, 15 years; sleep apnea
	67	M	Sore mouth	Lips, mouth, tongue, palate	Prednisone	AWD, 3 years
	61	M	Unknown	Lips through larynx	Not available	Not available
	56	M	Unknown	Nose, palate, pharynx	Resection	Not available

Van de Kerkhof and Van Baar [26]/1995	80	F	Pain, soreness, erythema, swelling	Lips	Betamethasone dipropionate	Partial symptomatic relief, f/u not available
	57	F	Sore throat	Gingiva, supraglottic larynx	Topical steroids	Gingival improvement, no f/u

Khan et al. [5]/1997	69	M	Hoarseness, dysphagia, hemoptysis	Left faucial pillar, hypopharynx, epiglottis, larynx	Beclomethasone spray, Corsodyl mouthwash	No recurrence, 20 months f/u

Noorily [25]/1997	67	M	Induration, erythema, crusting	Lower lip	Resection, primary closure	Not available

Smith et al. [4]/1999	59	M	Swelling, hoarseness, sore throat, erythema	Soft palate, gingiva, oropharynx, nasopharynx	None	Asymptomatic, 6 months f/u

Kaur et al. [24]/2001	47	M	Swelling, erythema, inflammation	Upper lip	Triamcinolone acetonide injections	No recurrence, 3 months f/u

Bharti and Smith [10]/2003	42	F	Pain, dysphagia	Buccal mucosa, palate	Topical and systemic antifungals, corticosteroids	Partial symptomatic relief, no regression of the disease, no f/u

Heinemann et al. [23]/2006	61	F	Pain, ulcerations, erythema, erosions	Tongue, lips, buccal mucosa, vulvae	Prednisolone, cyclosporin	Complete remission, 6 months f/u

Solomon et al. [2]/2008	60	F	Pain, sore throat, erosions, swelling, erythema	Gingiva, lips, ventral tongue, hard palate	Prednisone	Disease remission, f/u not available

Senol et al. [11]/2008	46	M	Inflammation, maceration, pruritic, eczema	Oral commissures, gingivobuccal mucosa, toe-webs, groins, preputium, perineum, umbilicus	Prednisolone	No recurrence, 1 year f/u

Najarian et al. [12]/2008	56	M	Pain, impetigo, bleeding	Lower lip	Topical and systemic glucocorticosteroid, cryotherapy	No recurrence, 6 months f/u

Pepper et al. [13]/2009	77	M	Atrophy, hyperkeratosis, erosion, pain	Commissure, lips, cheek	Tacrolimus, methotrexate, betamethasone mouthwash, CO_2_ laser, radiotherapy	Onset of squamous cell carcinoma, partial resolution of mucosal plasmacytosis

Puvanendran et al. [22]/2012	74	M	Ulceration, erythema	Uvula, hypopharyngeal wall	Resection	Lesion resolved, 6 months f/u

Gupta et al. [21]/2014	72	M	Difficulty in swallowing, soreness, burning sensation, sore throat, ulcerations, swelling, bleeding gums, erosions	Buccal mucosa, palate, tongue, pharynx, gingiva	Topical corticosteroids (betamethasone 1 mg, triamcinolone acetonide gel 0.1%), prednisolone, topical antifungals	Partial symptomatic relief, no regression of the disease, no f/u

Madhavarajan and Tighe [15]/2015	63	M	Pain, ulceration	Commissure, buccal mucosa, gingiva	No treatment	Spontaneous resolving, 6 months f/u

Cottom et al. [20]/2015	54	F	Erythema	Soft palate	Not available	Not available
	51	M	Erythema, ulceration, erosions	Border of tongue	Not available	Not available
	50	M	Inflammation	Gingiva	Not available	Not available
	67	M	Pain, bleeding, erosions	Gingiva	Not available	Not available
	83	F	Erosions	Gingiva	Not available	Not available
	65	M	Inflammation	Gingiva, hard palate	Not available	Not available
	72	M	Inflammation	Gingiva	Not available	Not available

Galvin et al. [14]/2016	68	F	Erythema, ulceration	Alveolar ridges, palate, buccal mucosa, gingiva	Oral fluconazole, chlorhexidine mouthwash, betamethasone cream and tablets	AWD, 7 years
	61	F	Sore mouth, ulceration, erythema, edema	Gingiva, buccal mucosa	Prednisolone, adalimumab	No recurrence, 18 months
	69	M	Oropharynx congestion	Gingiva, dorsum and borders of the tongue, soft palate, uvula	Mycophenolate mofetil, prednisolone	Partial symptomatic relief, no regression of the disease, no f/u

Trehan et al. [19]/2016	39	M	Ulceration, dysphagia, erosions	Lips, buccal mucosa, gingiva	Prednisolone	Good response, f/u not available

Arun et al. [17]/2017	13	M	Swelling, bleeding, erythema,	Upper lip, gingiva	Triamcinolone acetonide injections,	No recurrence, 1 year f/u

Liu et al. [18]/2017	18	F	Swelling, bleeding, erosions, pain	Lips	Methylprednisolone, dapsone, hydroxychloroquine, antibiotics	Symptoms decreasing, 6 months f/u

Gasparro et al. [6]/2019	78	F	Pain, erythema, ulcerations, erosions	Buccal mucosa	Injectable platelet-rich fibrin (i-PRF)	No complete healing, inflammation reduction, 6 months f/u

Shanahan et al. [16]/2019	62	F	Dry mouth, ulceration, swelling, dysphagia, hoarseness	Soft palate, buccal mucosa	Prednisolone, dapsone, mycophenolate mofetil	No recurrence of symptoms, 1 year f/u

AWD: alive with disease; F: female; f/u: follow-up; M: male. ^∗^Age at presentation (years).
